# Rheumatoid arthritis patients are not at increased risk for 30-day cardiovascular events, infections, or mortality after total joint arthroplasty

**DOI:** 10.1186/ar4385

**Published:** 2013-11-20

**Authors:** Kaleb Michaud, Edward V Fehringer, Kevin Garvin, James R O’Dell, Ted R Mikuls

**Affiliations:** 1 Omaha Veterans Affairs Medical Center, Omaha, Nebraska 68198-6270, USA; 2 National Data Bank for Rheumatic Diseases, Wichita, Kansas 68198-6270, USA; 3 University of Nebraska Medical Center, Omaha, NE 68198-6270, USA; 4 University of Nebraska Medical Center, 986270 Nebraska Medical Center, Omaha, NE 68198-6270, USA

## Abstract

**Introduction:**

Serious infection, cardiovascular disease, and mortality are increased in rheumatoid arthritis (RA). Whether RA affects the risk for these complications after total joint arthroplasty (TJA) is unknown, we hypothesize that it does. We compared the occurrence of 30-day postoperative complications and mortality in a large cohort of RA and osteoarthritis (OA) patients undergoing hip or knee TJA.

**Methods:**

Analyses included 7-year data from the Veterans Affairs Surgical Quality Improvement Program. The 30-day complications were compared by diagnosis by using logistic regression, and long-term mortality was examined by using Cox proportional hazards regression. All analyses were adjusted for age, sex, and clustering by surgical site. Additional covariates included sociodemographics, comorbidities, health behaviors, and operative risk factors.

**Results:**

The 34,524 patients (839 RA, 33,685 OA) underwent knee (65.9%) or hip TJA. Patients were 95.7% men with a mean (SD) age of 64.4 (10.7) years and had 3,764 deaths over a mean follow-up of 3.7 (2.3) years. Compared with OA patients, those with RA were significantly more likely to require a return to the operating room (odds ratio (OR), 1.45 (95% CI, 1.08 to 1.94), but had similar rates of 30-day postoperative infection, OR 1.02 (0.72 to 1.47), cardiovascular events, OR 0.69 (0.37 to 1.28), and mortality, OR 0.94 (0.38 to 2.33). RA was associated with a significantly higher long-term mortality; hazard ratio (HR), 1.22 (1.00 to 1.49).

**Conclusion:**

In this study of US veterans, RA patients were not at an increased risk for short-term mortality or other major complications after TJA, although they returned to the operating room more often and had increased long-term mortality.

## Introduction

Total joint arthroplasty (TJA) is among the most commonly performed elective surgeries, with more than a million total knee and hip arthroplasties completed in the United States in 2010 alone [1]. Overall charges in the United States for knee TJA in 2005 were US$8.85 billion [2,3]. With a rapidly aging population, coupled with increasing arthritis prevalence [4] and an escalation in the use of TJA [5-7], these numbers are expected to grow exponentially over the next few decades. Numerous investigations have detailed the substantial benefit of TJA in the treatment of end-stage arthropathy, benefits that include significant pain relief and improved functional status. Whereas general acceptance exists regarding the efficacy of TJA in end-stage arthritis, significant controversy surrounds the selection of patients who are most likely to benefit from joint-replacement surgery and, conversely, which patients are most likely to experience serious postoperative complications. Specifically, limited studies have examined the impact of TJA on overall mortality [8-15].

In addition to a focus on important clinical improvements in pain and function, most studies examining arthroplasty outcomes have exclusively involved patients with osteoarthritis (OA) because of its greater prevalence. The underrepresentation of patients with other forms of arthritis is highly relevant because TJA has been widely adopted as a therapeutic approach in other conditions, including rheumatoid arthritis (RA). Estimates suggest that approximately 18% to 25% of RA patients undergo TJA from disease onset more than 20 years [16,17]. RA patients have a higher prevalence than non-RA patients of selected comorbidities (that is, cardiovascular disease and serious infection) that could render this population at higher risk for serious postoperative complications after TJA. A recent systematic review of complications after TJA in RA and OA highlighted the need for more studies investigating these differences, most notably in infections, as their study hinted at increased risks in RA and mortality, for which most studies were underpowered [18].

In the present study, we assessed 30-day postoperative status, including cardiovascular events and infections, for a large population of patients with RA and OA undergoing initial TJA of the knee and hip. With a paucity of previous studies examining outcomes after TJA in RA, we also sought to identify patient factors associated with worse outcomes in this at-risk population.

## Methods

### Study subjects

Study subjects included patients in the Veterans Affairs Surgical Quality Improvement Program (VASQIP) database from years 1999 through 2006. VASQIP is a validated prospective quality-assurance and data-collection program that includes comprehensive data from surgeries performed under either general or epidural anesthesia [19,20]. An assessment of VASQIP has shown the data-collection efforts to be highly reliable, with >73% of data elements having substantial or almost perfect agreement with other surgical datasets [21]. As previously described, the database includes data on preoperative risk variables, intraoperative variables, and postoperative outcomes, including all-cause mortality [22]. The present study included surgical data from 106 collection sites from patients undergoing their first TJA of the hip or knee during the observation period. Total hip and knee arthroplasties were defined by using Current Procedural Terminology (CPT) codes of 27310 and 27447, respectively. Patients undergoing emergency surgery were excluded.

The VASQIP database was linked to outpatient and inpatient pharmacy dispensing data from the VA Pharmacy Benefits Management (PBM) database for the 1-year period preceding the index surgery. Linked PBM data also included all inpatient and outpatient ICD9 diagnoses for 1 year preceding surgery. Patients were classified as having RA if they had (a) at least one diagnostic code for RA (714.0) and (b) use of at least one biologic or nonbiologic DMARD during the 1-year preoperative period. This definition has previously been shown to have sensitivity/specificity exceeding 80% for identifying patients with RA [23]. Patients were categorized as having OA if they had at least one diagnostic code for OA (715.0 to 715.9), received no DMARDs 1 year before surgery, and lacked ICD9 codes for RA or alternative inflammatory arthritides (099.3, 555.9, 696.0, 710.0, 711.1*, 714.*, and 720.*). Patients who met only one of the two requirements for RA diagnosis, irrespective of OA diagnosis, were excluded from primary analysis. The Institutional Review Board and the Research & Development Committee at the Omaha VA Medical Center, in addition to the VASQIP Executive Committee, approved this protocol.

### Postoperative status and outcomes

Primary outcomes for the study were simplified into one continuous and several dichotomous variables: postoperative stay at hospital in days; return to the operating room; any complications [20]; any cardiovascular complications (cardiac arrest requiring CPR, myocardial infarction, cerebrovascular accident, pulmonary embolism, or deep-vein thrombosis); any infection complications (systemic sepsis, pneumonia, urinary tract infection, superficial surgical-site infection, and deep wound surgical-site infection); and mortality status.

Personnel in the VASQIP Data Center determined vital status by cross-matching these data with veteran death records in the Beneficiary Identification of Records Locator System (BIRLS) of the Veterans Benefits Administration. This system identifies all deaths, regardless of cause, affecting VA beneficiaries [21]. We defined short-term mortality status as mortality within 30 days of surgery. We obtained date of death through December 31, 2006.

### Predictors of postoperative outcomes

In addition to the specific type of TJA and arthritis diagnosis (RA versus OA), several potential determinants of postoperative outcomes included both preoperative and intraoperative factors [21]. Preoperative factors examined included age, gender, race, smoking status, diabetes mellitus, alcohol use, chronic obstructive pulmonary disease (COPD), patient-reported dyspnea, congestive heart failure (CHF), cerebrovascular disease, chronic glucocorticoid use, American Society of Anesthesiologists (ASA) class (dichotomized as healthy/mild versus severe/very severe), functional health status rated by dependency, and inclusive calendar 2-year of procedure categorized as 1999 through 2000, 2001 through 2002, 2003 through 2004, and 2005 through 2006.

Additional preoperative risk factors examined included seven selected laboratory abnormalities: blood urea nitrogen >40 mg/dl, creatinine >1.2 mg/dl, hematocrit ≤38%, platelet count ≤150,000/μl, serum sodium ≤135 or > 145 m*M*, or white blood cell count >11,000/μl.

Intraoperative factors examined included total operative time and anesthesia type. Potential predictor variables that were missing for more than 15% of patients were excluded (for example, do-not-resuscitate status, alkaline phosphatase, bilirubin, albumin, SGOT, prothrombin time, and partial thromboplastin time). In addition, we excluded possible preoperative predictor variables that were abnormal or prevalent in ≤0.9% of patients (for example, weight loss, diagnosis of disseminated malignancy, impaired sensorium, central nervous system tumor, wound infection, or open wound) after confirming that no significant prevalence differences existed between RA and OA.

Although VASQIP records if the patient is taking glucocorticosteroids (GCSs) before surgery, we thought greater understanding of the patient’s GCS history could be important for surgical outcomes. By using the VA PBM, we identified patients that were dispensed the GCSs dexamethasone, hydrocortisone, prednisolone, prednisone, or methylprednisolone at 30 days, 6 months, and 1 year before TJA surgery. We also identified patients who were dispensed GCS 10 days before surgery to account for possible perioperative stress dosing.

### Statistical analysis

Descriptive and comparative analysis was conducted by using the *t* test for continuous measures and a test of difference between two proportions for dichotomous measures. We conducted multivariable logistic and linear regressions with backward stepwise selection for analyzing each outcome of interest, with age, sex, and TJR type kept in every model. All regressions clustered patients by surgical site.

Short-term mortality analysis used a similar model with multivariable logistic regression of mortality status at 30, 180, or 365 days after surgery. Longitudinal mortality analysis was conducted by using Cox proportional hazards regression models, with the surgery date as the initial date for mortality risk until the date or death or censorship at December 31, 2006.

Data were analyzed by using Stata (College Station, TX, USA) version 11.1. Statistical significance was set at the 0.05 level; confidence intervals were established at 95%; and all tests were two-tailed.

## Results

### Patient characteristics

Of the 34,524 patients included in our primary analysis, only 839 (2.4%) had a qualifying RA diagnosis, with the remaining 33,685 (97.6%) having OA. Most surgeries were total knee arthroplasties (TKAs) (65.9%), and the average (SD) length of stay was 7.7 (7.3) days. Table 1 shows the characteristics of the patients by diagnosis. In comparison with OA patients, RA patients were younger, more likely to be female and Caucasian, and less likely to consume alcohol. No significant differences were found in smoking status. RA patients were less likely to have diabetes mellitus, although they were more likely to be rated with poor functional health.

**Table 1:** Characteristics of VA VASQIP patients receiving TKA or THA by OA or RA diagnosis

	**OA (*n*** **= 33,685)**			**RA (*n*** **= 839)**			***P***-**value**
	**Missing**	**Mean**	**SD**	**Missing**	**Mean**	**SD**	
**Demographics and comorbidity**							
Age (years)	0	64.49	10.72	0	62.19	10.55	<0.001
Male sex (%)	0	95.73		0	93.09		<0.001
Caucasian (%)	2,236	72.73		49	76.08		0.037
Alcohol consumption (%)	348	7.66		6	4.08		<0.001
Current smoker (%)	204	24.23		3	22.85		0.356
Diabetes mellitus (%)	204	16.88		3	12.20		<0.001
COPD (%)	1	9.67		1	10.74		0.301
Operation time (hours)	1	2.26	0.76	0	2.29	0.77	0.248
Low-dose aspirin (%)	0	38.09		0	27.89		<0.001
Poor functional health (%)	0	5.28		0	9.06		<0.001
Short of breath, dyspnea ≥2 (%)	274	11.17		8	9.63		0.162
General anesthesia (%)	0	69.39		0	70.92		0.343
Bleeding disorders (%)	206	1.34		4	1.44		0.812
Chemotherapy <30 days pre-op (%)	206	0.22		5	3.60		<0.001
CHF <30 days pre-op (%)	3	0.77		1	0.84		0.820
TIA history (%)	211	2.18		4	2.04		0.773
CVA/Stroke with neurologic deficit (%)	209	2.02		4	1.44		0.234
CVA/Stroke without neurologic deficit (%)	209	2.29		4	2.75		0.375
**GCS use before surgery**							
≤30 days	0	5.70		0	49.94		<0.001
≤0.5 year	0	11.68		0	62.22		<0.001
≤1 year	0	16.03		0	67.70		<0.001
At surgery interview (VASQIP)	0	1.16		0	41.12		<0.001
VASQIP or ≤1 year	0	16.42		0	69.25		<0.001
VASQIP and ≤1 year	0	0.77		0	39.45		<0.001
**Abnormal pre-op laboratory**							
Hematocrit ≤38% (%)	693	15.43		13	30.51		<0.001
White blood cells >11,000/μl (%)	890	3.85		16	10.45		<0.001
Platelets ≤150,000/μl (%)	1,175	5.71		20	3.30		0.003
Creatinine >1.2 mg/dl (%)	1,533	31.33		34	23.85		<0.001
Serum sodium ≤135 m*M* (%)	1,905	7.24		43	8.54		0.163
Serum sodium >145 m*M* (%)	1,905	1.63		43	1.01		0.165
Blood urea nitrogen >40 mg/dl (%)	2,990	0.93		80	0.79		0.689

Several DMARDs were taken by RA patients before surgery, including methotrexate (58.6%), hydroxychloroquine (40.4%), biologics (24.0%), and sulfasalazine (23.2%). By definition of diagnosis group, none of the OA patients was taking a DMARD. RA patients were much more likely to be taking GCS and chemotherapy and less likely to be taking low-dose aspirin before surgery (Table 1).

A large number of patients were missing several preoperative laboratory measures, with only 10,673 patients (10,387 OA and 286 RA) having all 13 values available (Additional file 1: Table S1). This group was studied in a separate analysis that included all laboratory values, as abnormalities may have been associated with other comorbidities or alerted the surgeon to take precautionary measures. Of the preoperative laboratory values used in the primary analysis, 7.3% of patients were missing at least one of the seven values examined.

### TJA complications

We examined the association of all covariates on several surgical-complication outcomes (Table 2) in multivariable regressions. A post-operative cardiovascular event occurred in 2.0% of patients, and although it was associated with risk factors such as age, time of operation, COPD, dyspnea, preoperative wound or infection, transient ischemic attack (TIA) history, and creatinine >1.2 mg/dl clearance, factors including male sex and RA versus OA were not significantly associated (Table 3). Even more covariates were linked with postoperative infections, including the receipt of diabetes treatments, female sex, and poor functional health, although again, RA diagnosis had no association and, unexpectedly, GCS dispensing in the month before TJA was protective (OR, 0.75 (95% CI, 0.58 to 0.96)). Deep-wound surgical infections had similar associations, although a positive association was noted with dispensing in the 6 months before surgery (OR, 1.86 (1.33to 2.60)). Considering all post-TJR complications, neither an association with RA diagnosis nor one with the specific target joint being replaced was found, as shown in Table 3.

**Table 2:** Surgical outcomes of patients receiving TKA or THA by OA or RA diagnosis

	**OA (*n*** **= 33,685)**			**RA (*n*** **= 839)**			***P *** **value**
	**Missing**	**Mean**	**SD**	**Missing**	**Mean**	**SD**	
**Outcome**							
Postoperative stay (days)	130	7.77	7.31	7	7.77	5.79	0.994
Return to operating room (%)	0	3.06		0	4.53		0.015
Any complications? (%)	0	7.04		0	6.67		0.679
**Any PO CV event (%)**	0	2.04		0	1.31		0.139
**Any PO infection (%)**	0	4.15		0	4.17		0.972
Superficial surgical-site infection (%)	0	1.10		0	1.19		0.798
Deep wound surgical-site infection (%)	0	0.65		0	0.36		0.291
**TJR type**							
Hip (%)	0	34.19		0	31.35		0.087
Knee (%)	0	65.80		0	68.70		0.087

**Table 3:** **Multivariable logistic-regression model results for complications associated with hip or knee TJA**^a^

**Variable**	**Any post-op cardiovascular event**	**Any post-op infection**	**Deep wound surgical-site infection**	**Any complications**	**Post-op stay (β in days)**	**Return to operating room within 30 days**	**Mortality**^**b**^
**RA versus OA**^**c**^	0.66 (0.35, 1.24)	1.14 (0.80, 1.62)	0.35 (0.12, 1.08)	1.07 (0.80, 1.43)	-0.18 (-0.73, 0.38)	1.41 (1.05, 1.90)	1.22 (1.00, 1.49)
**Age**^**c**^	1.03 (1.02, 1.04)	1.03 (1.02, 1.03)	0.99 (0.98, 1.01)	1.02 (1.02, 1.03)	0.10 (0.08, 0.12)	1.00 (0.99, 1.01)	1.06 (1.05, 1.06)
**Male sex**^**c**^	1.04 (0.66, 1.64)	0.68 (0.53, 0.87)	0.77 (0.46, 1.29)	0.8 (0.67, 0.95)	-1.06 (-1.51, -0.61)	1.05 (0.78, 1.42)	^d^
**Hip versus knee TJA**^**c**^	0.88 (0.73, 1.05)	1.10 (0.96, 1.26)	0.96 (0.68, 1.35)	1.10 (0.99, 1.23)	0.62 (0.33, 0.90)	1.38 (1.17, 1.63)	1.24 (1.16, 1.33)
**Operation Time (in hours)**	1.10 (1.00, 1.22)	1.11 (1.01, 1.23)	1.22 (1.04, 1.43)	1.16 (1.08, 1.24)	1.10 (0.75, 1.45)	1.23 (1.13, 1.33)	1.30 (1.19, 1.41)
**General anesthesia**		1.17 (1.00, 1.39)	1.43 (0.98, 2.10)	1.17 (1.04, 1.32)			
**Calendar year (1998–2006)**		1.03 (1.00, 1.07)			-0.28 (-0.37, -0.18)		
**Caucasian race**					-0.98 (-1.68, -0.28)		
**Alcohol consumption**				1.19 (1.02, 1.39)			1.23 (1.09, 1.38)
**Current smoker**	0.78 (0.62, 1.00)		1.72 (1.30, 2.26)				1.57 (1.45, 1.71)
**Poor functional health**		1.48 (1.20, 1.81)	1.66 (1.07, 2.58)		1.62 (0.79, 2.46)	1.42 (1.13, 1.78)	
**Short of breath, dyspnea ≥2**	1.53 (1.22, 1.93)			1.27 (1.10, 1.47)		1.36 (1.10, 1.66)	1.20 (1.09, 1.32)
**COPD history**	1.27 (1.01, 1.60)	1.81 (1.52, 2.16)		1.47 (1.27, 1.70)	0.54 (0.22, 0.87)		1.45 (1.32, 1.60)
**Bleeding disorder**	2.34 (1.63, 3.37)	1.83 (1.28, 2.62)					
**Pre-op open wound/infection**	3.02 (1.28, 7.13)	2.46 (1.32, 4.60)		2.73 (1.62, 4.61)		2.86 (1.53, 5.35)	1.91 (1.20, 3.04)
**TIA history**	2.03 (1.46, 2.81)			1.41 (1.11, 1.79)			
**Stroke history w/impairment**					1.23 (0.49, 1.96)		
**Stroke history w/o impairment**						1.49 (1.00, 2.21)	1.21 (1.02, 1.44)
**CHF <30 days pre-op**				1.53 (1.02, 2.29)	2.54 (0.61, 4.47)		1.55 (1.22, 1.96)
**Impaired sensorium**							2.30 (1.61, 3.28)
**Diabetes: oral treatment**		1.18 (1.02, 1.37)	1.59 (1.14, 2.21)	1.2 (1.05, 1.37)			
**Diabetes: insulin treatment**		1.36 (1.09, 1.69)	2.48 (1.65, 3.73)	1.41 (1.18, 1.70)			
**Diabetes: oral or insulin**					0.40 (0.16, 0.64)		
**Chemotherapy <30 days preop**							1.72 (1.19, 2.49)
**Low-dose aspirin**		0.76 (0.64, 0.90)	0.47 (0.33, 0.67)	0.77 (0.68, 0.88)		0.71 (0.62, 0.82)	0.65 (0.60, 0.70)
**White blood cells >11,000/μl**							1.35 (1.16, 1.56)
**Creatinine >1.2 mg/dl**	1.20 (1.00, 1.44)						
**Hematocrit ≤38%**		1.21 (1.04, 1.42)		1.25 (1.12, 1.40)			1.47 (1.36, 1.58)
**Serum sodium >145 m** ***M***		1.64 (1.07, 2.52)					
**GCS (30 days)**		0.75 (0.58, 0.96)		0.82 (0.69, 0.97)			
**GCS (6 months)**			1.86 (1.33, 2.60)				
**GCS (1 year)**					0.45 (0.18, 0.72)		
**GCS (VASQIP)**							1.52 (1.26, 1.82)
**Constant**					557.9		

^a^Except where noted, odds ratios (95% CI).

^**b**^Cox proportional hazards regression, hazard ratios (95% CI).

^**c**^Variable was forced into all models.

^**d**^Not significant and removed for not meeting proportional hazard requirement.

CHF, congestive heart failure; COPD, chronic obstructive pulmonary disease; GCS, glucocorticosteroid; OA, osteoarthritis; RA, rheumatoid arthritis; TIA, transient ischemic attack. TJA, total joint arthroplasty.

In similar analysis, length of hospital stay in days during TJA was increased by a day for each decade of age, another day for each hour of surgery, and a half-day for having hip (versus knee) surgery, and was decreased a day for men and for every 3 calendar years. No association was found between having RA with this length of hospital stay, although having CHF, stroke, diabetes, COPD, GCS use in the past year, and poor functional status were all associated with an increased length of stay. When looking at any return to the operating room within 30 days of TJA, RA patients were 40% more likely to return to the operating room (OR, 1.41 (1.05 to 1.90)), as were those receiving hip versus knee surgery (OR, 1.38 (1.17 to 1.63)). No analyses showed an association of removing or separating patients who had GCS use within 10 days before surgery from those patients who took GCS in larger time windows beforehand.

We repeated these analyses for the subset of 10,673 patients with full laboratory data. Except for the length of stay, for which RA patients had a significantly decreased stay of -0.97 (-1.82 to -0.12) days, none of the other TJA outcomes had appreciable differences in magnitude between RA and OA.

### TJA mortality

In total, 3,764 deaths over a mean follow-up of 3.7 (2.3) years occurred in the analysis cohort. Mortality was initially examined in models similar to other complications, although at three discrete time points after TJA. At no point up to 1 year was there a statistically important difference between RA and OA, although after covariate adjustment, a trend was noted toward an increase in mortality for RA over time: 30-day OR 0.94 (0.38 to 2.33), 6-month OR, 1.25 (0.72 to 2.19), and 1-year OR, 1.30 (0.87 to 1.96).

**Figure 1:**
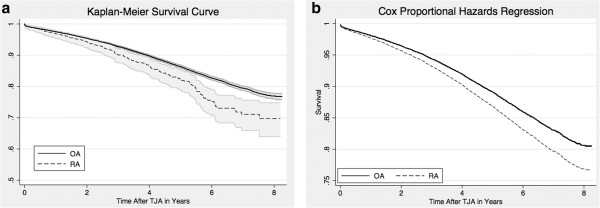
**Mortality after total joint arthroplasty by rheumatoid arthritis (RA) and osteoarthritis (OA) diagnosis through (A) Kaplan-Meier curves with 95% confidence intervals and (B) Cox proportional hazards regression model adjusted for 16 covariates listed in Table **3**.**

When examining mortality over time continuously until 2007 by using Cox proportional hazards regression, RA patients had a hazard ratio (HR) of 1.22 (1.00 to 1.49). Associated covariates were similar to the prior dichotomous mortality analysis, and the full model is shown in Table 3. The Kaplan-Meier curve and adjusted survival function for this full model by diagnosis are shown in Figure 1.

## Discussion

Covering 8 years in 106 VA surgical sites, our sizable study analyzed and compared the experiences of 34,524 OA and RA patients receiving their initial total knee- or hip-replacement surgery. No associations with RA versus OA were found for surgical complications, including cardiovascular and infectious events occurring within 30 days of the surgery. Whereas we found no association of RA with length of hospital stay, patients were significantly more likely to return to the operating room within 30 days of their TJA. Mortality remained similar for RA and OA after TJR, except for the increase in RA when we allowed a time horizon beyond 1 year after surgery.

Although our study is the second to use the VASQIP database to examine the impact of patient and surgery covariates on TKA and THA outcomes, the prior study by Weaver *et al*. [19] reported on data from 1991 to 1997, all before the current study’s start date of 1999, and did not have several of the measures we analyzed, including RA diagnosis. They did not find alcohol use to be significantly associated with postsurgical complications, in contrast with results from our study, which is consistent with a more recent analysis of VASQIP [24]. We previously analyzed a portion of the current data, comparing outcomes for total shoulder arthroplasty with TKA and THA, which provides an alternative view of the current study [25]. Both the study of Weaver *et al*. and our study complement each other and provide useful tools for determining the risk of postoperative complications by an individual patient’s characteristics, which is especially useful as the number of TJAs increases in the aging general and VA population.

Whereas the overall rates of TJA increase, the rates in RA patients have been decreasing in past decades, likely because of improvements in treatments, although TJA still represents an important outcome in the failure to control progressive joint damage from RA [8,16,26]. Although patients undergoing TJA have been reported to have a greater risk of surgical complications, our study did not find evidence of this. Whether orthopedic surgeons take greater preventative care when operating on RA patients or whether they postpone this often-elective procedure to a time (or indefinitely) with the least amount of RA activity, the current study’s observational data cannot address this, and we are left speculating.

A few studies have shown increased risk of TJA joint infection in RA versus OA patients [27,28], although all had a much longer follow-up in comparison to that of the current study, which found no statistically significant increased risk of 30-day infection in RA patients. Bongartz *et al*. [28] found 1.2% of 657 TJAs in RA had infection within 30 days, which is the same rate that we found for our superficial site infection, but much higher than our deep-wound site infection (0.4%). Although we also controlled for age, sex, function, comorbidities, prior infection, and more, we found no increase in RA infections; this difference may be because our population all had primary surgery, whereas they found most of their infections in TJA revision [28]. We did find contradictory results in the impact of GCS on overall postoperative infections, which were protective when used less than 30 days before surgery (OR, 0.75 (0.58 to 0.96)), and deep-wound infections, which were associated with GCS when dispensed less than 6 months beforehand (1.86 (1.33 to 2.60)). It is difficult to interpret this without access to dosage of GCS, but it is likely that a bias exists for near-TJA surgical date prescription compared with longer-term prescriptions, which have been shown to have a near-exponential dose-time impact on future infections [29].

The systematic review by Singh and colleagues [10] addressed mortality after TKA and THA. They found no significant difference in 30-day and 90-day mortality between RA and OA patients, although they indicated that this may be due to the small number of studies reporting these data (for example, a total of four deaths in 481 RA patients at 90 days). We suspect that all 20 of their reported 30-day mortality events (of 7,174 RA patients) came from one surgical site’s records during 1969 through 1997 [13,15], all before the dates of the current study. Similar power issues are found with the Ravi *et al*. [18] systematic review that showed no difference in mortality.

Domsic *et al.*[30] looked at mortality during the hospital stay for TKA and THA by using the large Nationwide Inpatient Sample from 1993 through 2006. They reported similar results that mortality in RA was no different from that in their non-lupus and non-RA controls, although in several multivariable subanalyses, they found RA to be protective of mortality for all elective surgeries: OR, 0.58 (95% CI, 0.41 to 0.82), and for all TKA, OR, 0.64 (0.44 to 0.92). The reasons for these findings are not clear, although their average length of hospital stay was 2 to 3 days shorter than ours, and they had a greater RA versus OA age difference.

A limitation of our study is the relatively low ratio of RA to OA patients (2.4%), which reduced our power to detect less-robust associations, although this is likely related to the substantially increased number of men in the VA system, compared with the average clinical population, who are less likely than women to develop RA. Recognizing these limitations, our study results are still based on a considerable number of surgeries, making it one of the largest studies of its kind to date. Although important RA covariates of clinical disease activity were not available, our study did include the possible impact of RA treatments like GCS, along with comorbidities that are often associated with worse disease and infections [29]. Our data unfortunately did not record the reason for a return to the operating room, and we must speculate whether this was due to dislocations or other causes.

This highlights another limitation, that our study relies up the patient diagnosis and classification of a large registry and that we were unable to validate the reported outcomes or review the patient records, although this is true of almost all administrative data studies.

The important risk factor of obesity was not available in our data. The study by Suleiman *et a*l. [31] did not find any increased rates of perioperative complications related to obesity in patients undergoing TKA and THA, although they did not investigate by arthritis diagnosis. With RA, we found an expected increase in TJA and comorbidity with obesity, although we also found increased TJA in those who were underweight and a decrease in mortality in those who were overweight [32]. A similar protective effect of obesity was found, even after taking RA diagnosis into account in the Clement *et al.* study of mortality after TKA [9], although by keeping body mass index a continuous measure, they were unable to indicate whether this was driven by those who were underweight. A similar issue may have occurred in the Olmsted County study that found only an increase in TJA with obesity [8].

## Conclusions

Although we expected to find greater risk of complications in RA patients, especially because TJA is reserved for those with the most-severe disease and therefore greater risk, our results did not support this in the short term. Although preoperative risk assessment remains critical in mitigating postoperative complication, including infection and cardiovascular events, our results would suggest that the diagnosis of RA alone should not preclude consideration of this important intervention. Future studies are needed to elucidate the impact of specific treatments, obesity, and any additional steps surgeons may take with RA patients around these increasingly important surgical procedures.

## Abbreviations

ASA: American Society of Anesthesiologists; BIRLS: Beneficiary Identification of Records Locator System; CHF: Congestive heart failure; COPD: Chronic obstructive pulmonary disease; CPR: Cardiopulmonary resuscitation; CPT: Current procedural terminology; CV: Cardiovascular; CVA: Cerebrovascular accident; DMARD: Disease-modifying antirheumatic drug; GCS: Glucocorticosteroid; HR: Hazard ratio; ICD9: International Statistical Classification of Disease and Related Health Problems, Ninth Revision; OA: Osteoarthritis; OR: Odds ratio; PBM: Pharmacy Benefits Management; PO: Postoperative; RA: Rheumatoid arthritis; SD: Standard deviation; SGOT: Serum glutamic oxaloacetic transaminase; THA: Total hip arthroplasty; TIA: Transient ischemic attack; TJA: Total joint arthroplasty; TKA: Total knee arthroplasty; VA: Veterans Affairs; VASQIP: Veterans Affairs Surgical Quality Improvement Program.

## Competing interests

EF receives royalties for the development of shoulder prostheses. The remaining authors declare that they have no competing interests.

## Authors’ contributions

TM and JO acquired the data. KM and TM conceived and designed the study. EF and KG contributed to interpretation of the data. All authors contributed to, edited, read, and approved the final manuscript.

## Supplementary Material

Additional file 1: Table S1 Excluded laboratory characteristics of VA VASQIP patients receiving TKA or THA by OA or RA diagnosis. Click here for file
