# Supplementary material for: Rheumatoid arthritis patients are not at increased risk for 30-day cardiovascular events, infections, or mortality after total joint arthroplasty
Source: Arthritis Res Ther. 2013 Nov 20;15(6):R195. doi: 10.1186/ar4385 (PMC3978488; doi:10.1186/ar4385)
Supplement: Additional file 1: Table S1 — Excluded laboratory characteristics of VA VASQIP patients receiving TKA or THA by OA or RA diagnosis. [file ar4385-S1.docx]

**Additional file 1: Table S1. Excluded laboratory characteristics of VA VASQIP patients receiving TKA or THA by OA or RA diagnosis.**

|  | **OA (N=33,685)** | | **RA (N=839)** | | **P-Value** |
| --- | --- | --- | --- | --- | --- |
| **Lab** | **Missing** | **%** | **Missing** | **%** |  |
| *Alkaline phosphatase >125 mU/ml* | *15489* | *6.37* | *261* | *10.73* | *<0.001* |
| *Total bilirubin >1.0 mg/dl* | *14648* | *7.41* | *237* | *3.16* | *<0.001* |
| *Serum albumin <3.2 g/dl* | *13043* | *1.08* | *202* | *8.63* | *<0.001* |
| *Prothombin time >13.3 s* | *12298* | *22.78* | *334* | *26.73* | *0.036* |
| *Prothombin time >35 s* | *12317* | *6.05* | *330* | *7.47* | *0.187* |
| *Serum glutamic oxaloacetic test >40 mU/ml* | *15164* | *7.36* | *218* | *4.35* | *0.004* |
